# Factor Structure and Measurement Invariance of Youth Psychopathic Traits Inventory-Child Version in Chinese Children

**DOI:** 10.3389/fpsyg.2019.02550

**Published:** 2019-11-27

**Authors:** Fen Ren, Qing-peng Zhang, Mingshu Li, Jie Luo, Jiaxin Deng, Xintong Zhang, Meng-Cheng Wang

**Affiliations:** ^1^School of Education and Psychology, University of Jinan, Jinan, China; ^2^Department of Sociology, School of Public Administration, Guangzhou University, Guangzhou, China; ^3^The Center for Psychometrics and Latent Variable Modeling, Guangzhou University, Guangzhou, China; ^4^Department of Psychology, Guangzhou University, Guangzhou, China; ^5^School of Education Science, Guizhou Normal University, Guiyang, China; ^6^The Key Laboratory for Juveniles Mental Health and Educational Neuroscience in Guangdong Province, Guangzhou University, Guangzhou, China

**Keywords:** psychopathic traits, Youth Psychopathic Traits Inventory-Children Version, bifactor model, measurement invariance, Chinese children

## Abstract

The primary goal of the present study was to examine the latent factor structure and measurement invariance (MI) of the Youth Psychopathic Traits Inventory-Child Version (YPI-CV) in a sample of Chinese children. 299 school children (aged 9–12, 47.3% female) completed the Chinese version of the YPI-CV, and their parents completed a different measure of psychopathic traits, as well as ones for other measures: the Child Problematic Traits Inventory (CPTI), the Strength and Difficulty Questionnaire (SDQ), and the Social Competence – Parent Version (SCPV). Results showed that a bifactor model at item level fit the data best and was invariant across gender. Specifically, the general psychopathy factor influenced the 18 items strongly, suggesting that the YPI-CV is unidimensional rather than multidimensional. Overall, findings suggest that the bifactor structure of the YPI-CV should be used when examining relationships with outcome variables in Chinese children, with a focus on the total score of the YPI-CV, while factor scores should explain with caution.

## Introduction

Psychopathic personality refers to a constellation of personality traits, such as callousness, manipulativeness, egocentricity, impulsivity, and seeking stimulation (e.g., Cleckley, 1988; Hare, 2003). Recent models of psychopathy have conceptualized it into three dimensions: affective callous-unemotional (CU) traits, interpersonal-manipulative traits, and impulsive and irresponsible behaviors (Cooke and Michie, 2001). These psychopathic traits have been considered as a significant predictor of violence and criminality among adults (Caputo et al., 1999; Hare, 2003; Douglas et al., 2006). Recent research has extended this concept to adolescents and children as well (e.g., Barry et al., 2000; Forsman et al., 2008; Bijttebier and Decoene, 2009; Salekin and Lynam, 2010; Salekin, 2017), with the important implication that understanding psychopathic traits in youth can help us to gain insight into the different pathways that lead toward severe antisocial behaviors (Frick, 2009). To date, studies of psychopathic traits in youth have generally yielded similar findings to studies looking at those in adults, in terms of stability, relations to conduct problems and aggression, and emotional and cognitive correlates (for reviews see, e.g., Kotler and McMahon, 2005; Lynam and Gudonis, 2005; Salekin, 2017).

Several instruments have been developed to assess psychopathic traits in youth and children. Among them, the Youth Psychopathic Traits Inventory (YPI; Andershed et al., 2002; Skeem and Cauffman, 2003; Muñoz et al., 2019), a four-point Likert scale of 1 (does not apply at all) to 4 (applies very well) consisting of 50 items subdivided into 10 subscales, has been considered particularly favorable in several studies (e.g., Kotler and McMahon, 2005; Vaughn and Howard, 2005). It has been argued that it is a superior or comparable to other instruments in measuring psychopathic traits, such as the Antisocial Process Screening Device (APSD; Frick and Hare, 2001; Poythress et al., 2006; Colins et al., 2014b) and the Psychopathy Checklist: Youth Version (PCL:YV; Forth et al., 2003; Chauhan et al., 2014).

The YPI applies to a wide age range because the items in the adolescent version (Andershed et al., 2002) and child version (Van Baardewijk et al., 2008) are almost identical. Consistent with recent theoretical models, a three-factor structure was supported (e.g., Andershed et al., 2002; Larsson et al., 2006; Wang et al., 2017). The internal consistencies of the three YPI dimensions and the total scores have been good to excellent, with Cronbach’s α for total score ranging from 0.87 to 0.92, the CU dimension from 0.74 to 0.81, the Grandiose-Manipulative from 0.82 to 0.90, and the Impulsive-Irresponsible from 0.68 to 0.85 (Andershed et al., 2002; Skeem and Cauffman, 2003; Larsson et al., 2006; Andershed et al., 2007). The validity of the YPI also has been demonstrated by significant correlations with the PCL:YV (*r* = 0.29–0.48; Skeem and Cauffman, 2003; Dolan and Rennie, 2006; Andershed et al., 2007). Furthermore, YPI scores were found to predict subsequent institutional infractions and violence, especially for the lifestyle/antisocial elements (Dolan and Rennie, 2006, 2007). Additionally, the YPI has shown correlations with indices of previous antisocial behavior (Andershed et al., 2002; Skeem and Cauffman, 2003; Larsson et al., 2007) and can be used to identify a severe and aggressive subgroup of antisocial adolescents (Andershed et al., 2002; Dolan and Rennie, 2006).

For most studies, however, the lengthy 20-min administration time of all 50 items may not be necessary. Through a stepwise selection process, a short version of the YPI (YPI-SV) was developed, with only 18 items, by Van Baardewijk et al. (2010), with six items for each of the three factors. Items were also shortened to save time. For example, “I don’t let my feelings affect me as much as other people’s feelings seem to affect them,” has been changed to, “Feelings are less important to me than they are for others.” The YPI-SV showed a high convergence with the original 50-item instrument (*r_*s*_* = 0.95) and had similar correlations with external criterion measures of conduct problems (e.g., Wang et al., 2017). The reliability measured using Cronbach’s α for the total score is 0.80–0.85 in different samples. In sum, the short version is a practical and valid alternative to the original YPI.

In recent past, several self-report assessment tools of psychopathic traits were introduced to Chinese adults (Shou et al., 2017; Wang et al., 2018b) and adolescents (Wang et al., 2015, 2017). For example, the Chinese version of the YPI and YPI-SV were validated in a large sample of adolescents and findings demonstrated good to excellent reliability, factorial validity and construct validity in this population (Wang et al., 2017). However, no Chinese version of an assessment tool suitable for children is available at present, particularly in a self-report format. Past studies have shown that psychopathic traits can be observed at very young ages (Kimonis et al., 2016). The Youth Psychopathic Traits Inventory-Child Version (YPI-CV) was developed to create an age-appropriate brief version of the YPI that matches the cognitive, emotional, and verbal development and social realities of 9- to 12-year-olds. Eighteen items were selected from the original YPI 50 items based on the discrimination (more than 0.40) and difficulty parameters (around 0.50) (Van Baardewijk et al., 2010).

On the other hand, the factor structure of the YPI and YPI-SV has been an uncertainty topic in recent years. In the original version of YPI, a three-factor model was tested and proved as an acceptable model (Larsson et al., 2006; Wang et al., 2017). Recently, a bifactor model for the YPI-SV has been supported in several previous studies conducted by Wang et al. (2017) and others (Shou et al., 2017; Wang et al., 2018b). Since Patrick et al. (2007) assumed a higher-order model of psychopathy, it is possible that a single construct – the one-factor model may also be accepted. Therefore, we considered to compare the three possible models in the present study and choose the best fit model to test the measurement invariance (MI).

An interesting test of the construct validity of an instrument referring to test invariance had to do with the investigation of potential latent means difference across subgroups to make sure whether these difference can be replicated under the same construct (Slof-Op’t Landt MC et al., 2009). Previous studies have already checked MI of youth psychopathy in different demographic backgrounds, such as genders (Andershed et al., 2002), and age groups (Pihet et al., 2014). As a new measurement tool of youth psychopathy, in order to examine whether boys and girls intercept the questions of YPI-CV similarly, such MI tests must be established before gender comparison.

### The Current Study

The overarching goal of the present study was to examine the validity of the Chinese version of the YPI-CV. Specifically, we assessed the latent structure of the YPI-CV using confirmatory factor analysis (CFA). Three competing models were examined: a one-factor model with all 18 items loaded on a single factor, a three-factor model with all items loaded on three correlated factors, and a bifactor model with each item loaded on one of the three factors, as well as on a general factor. Based on prior findings (e.g., Wang et al., 2017), we expected that the bifactor model would fit the data best. Moreover, the MI (i.e., configural, metric, and scalar invariance) of the YPI-CV would be examined. We expected a good to acceptable model fit across genders. Finally, to test the convergent and divergent validity, parent-reported psychopathic traits on the Child Problematic Traits Inventory (CPTI; Colins et al., 2014a; Wang et al., 2018a), social competence and psychosocial functioning of the children were assessed as criteria variables. It was hypothesized that low to moderate correlations between the YPI-CV scores and criteria variables would be found.

## Materials and Methods

### Participants

A total of 299 participants from four grades were recruited from a public primary school in the city of Guangzhou in Guangdong Province of China through a primary subject pool. Every child completed the questionnaire between two classes break under construction from trained graduate students. The questionnaires for parents with consent forms were taken home by children in envelopes and brought them back within 2 days. The mean age of the total sample was 10.44 years (*SD* = 0.89; range = 9–12 years), and 47.3% were female.

The ages of the parents ranged from 29 to 63 years old. The income levels of the families were divided into six major categories; 61.5% (*N* = 184) of the families were at a high level (>8,000 RMB per month). The education levels of parents were as follows: 40 (13.4%) junior high school or below, 51 (17.1%) senior high school, 39 (13.0%) undergraduate, 73 (24.4%) graduate, 78 (26.1%) doctoral degree, and the remaining 18 (6.0%) unknown (see Table 1 for more information).

**TABLE 1:** Sample demographics statistics.

	**Category**	**Frequency**	**Percentage**
Gender	Male	158	52.7
	Female	141	47.3
Age(male)	9	16 (5)	5.4
	10	140 (72)	46.8
	11	105 (59)	35.1
	12	21 (13)	7.0
	Unreported	17	5.6
Education background of parents	Junior high school and below	40	13.9
	Senior high school	51	17.1
	Undergraduate	39	13.0
	Graduate	73	24.4
	Doctorate	78	26.1
	Unreported	18	6.0
Family income per month in RMB	<4,000	50	16.7
	4,000–8,000	53	17.7
	8,000–12,000	108	36.1
	12,000–16,000	38	12.7
	16,000–20,000	19	6.4
	>20,000	19	6.4
	Unreported	12	4.0
Total		299	100

## Measures

### Youth Psychopathic Traits Inventory – Child Version (YPI-CV)

The YPI-CV consists of 18 items, with six items included in each subscale. Each child completed the questionnaire using a four-point scale from 1 (does not apply at all) to 4 (applies very well). Items of the original YPI-CV were translated into Chinese and back-translated to English by the authors (native Chinese speakers) who reached an agreed-upon translation of each item. Similar processes were used to develop the Chinese versions of the YPI and YPI-SV (Wang et al., 2017). The internal consistency reliability of the total score measured by Cronbach’s α is 0.725 (Novick and Lewis, 1967).

### Child Problematic Traits Inventory (CPTI)

The CPTI was originally developed with the intention that the teacher assesses psychopathic traits in 3- to 12-year-olds. The CPTI items were developed using a theory-driven approach based on the three-factor model of psychopathy (Cooke and Michie, 2001): Grandiose-Deceitful (GD; eight items), CU(10 items), and Impulsive-Need for Stimulation (INS; 10 items). The three factors showed acceptable internal consistency and external validity, with expected correlations with theoretically relevant constructs (e.g., fearlessness; Colins et al., 2014b). The Chinese version of the CPTI was also validated and showed excellent internal consistency and construct validity (Wang et al., 2018a). In this study, internal consistency was good for the CPTI total (α = 0.918) and three subscales (α = 0.781 for family rated GD, α = 0.845 for CU, and α = 0.795 for INS).

### Strength and Difficulty Questionnaire (SDQ)

The SDQ (Goodman, 1997; Muris et al., 2004; Ullebø et al., 2011) and its Chinese version (Du et al., 2008; Liu et al., 2013) are screening instruments for the psychosocial functioning of children and adolescents^1^. The questionnaire consists of five subscales of five items covering emotional problems, conduct problems, hyperactivity problems, peer problems, and prosocial behavior (Essau et al., 2012). Responses to each item use a three-point ordinal Likert format and can be answered with “not true,” “somewhat true,” or “certainly true.” Responses can be rated from 1 to 3 for negatively worded items and 3 to1 for positively worded items. Therefore, higher scores indicate more problematic attributes. Scores are generated for each subscale (possible range: 3–15), and scores for all subscales except the prosocial behavior scale are summed to form a total difficulties score (possible range: 20–60). The prosocial behavior subscale measures the child’s ability to act prosocially, independent of the difficulties measured by the other subscales. In this sample, the Cronbach’s α was 0.754 for the SDQ total difficulties scores, indicating good reliability (Osburn, 2000; Ponterotto and Ruckdeschel, 2007).

### Social Competence – Parents Version (SCPV)

Parents also completed the SCPV, as developed by the Conduct Problems Prevention Research Group (Conduct Problems Prevention Research Group [Cpprg], 1995). This scale consists of 12 items that assess a child’s positive social behaviors, including emotion regulation, prosocial behaviors, and communication skills. Parents are asked to rate how well each of the twelve statements describes their child on a five-point Likert scale, from 1 (not at all) to 5 (very well). In previous studies with elementary school-aged children, this measure has yielded one total and two reliable subscales: emotion regulation and prosocial/communication skills (Conduct Problems Prevention Research Group [Cpprg], 1995; Corrigan, 2003; Foley and Weinraub, 2017; Milligan et al., 2017). Internal consistencies are from 0.76 to 0.82 for emotion regulation, 0.74–0.84 for prosocial/communication skills, and 0.84–0.89 for the total score (Corrigan, 2003; Milligan et al., 2017). Cronbach’s αs for the current sample were 0.90, 0.78, and 0.88 for the total score, emotion regulation, and prosocial/communication, respectively, indicating good reliability.

## Procedure

The children completed the self-report questionnaires during a classroom session under the supervision of a specially trained research assistant (a masters-level student). The administration time was approximately 45 min. Children could ask the research assistant for clarification if they did not understand the question. Before the administration of the self-report questionnaires, the children’s parents or legal guardians provided their written informed consent, and the children provided assent to participate.

Parental questionnaires and consent letters were placed in envelopes and taken home by the children. The parents were instructed to return the questionnaires in a sealed envelope to their teacher within 2 days. The study was reviewed and approved by the Human Subjects Review Committee at Guangzhou University (Review No. 20141008).

### Data Analysis Strategy

#### Stage 1: Factor Structure

In line with previous studies (e.g., Zwaanswijk et al., 2017), three different factor models would be tested using CFA. Both three-factor and bifactor models were recommended in many studies to present the latent structure of youth psychopathy construct (Neumann et al., 2006; Neumann and Pardini, 2014; Zwaanswijk et al., 2017). Besides, Patrick et al. (2007) assumed a higher-order model of psychopathy, and the YPI-CV was computed the total score in practical usage; thus, we took a one-factor model into consideration in the present study based on simple construct principal. CFAs were performed at the item level to examine the best-fit model for the YPI-CV using the robust weighted least squares with mean and variance adjustment (WLSMV) estimator to estimate all parameters (Flora and Curran, 2004; Beauducel and Herzberg, 2006). At the item level, the response scale was an ordinal scale with four response options. The item level was treated as categories, and multiple-group confirmatory factor analysis (MCFA) was used to test MI(Dolan, 1994; Roger and Jenn, 2004) between the two gender groups.

Model fit was evaluated by chi-square (WLSMV χ*2*) and associated degrees of freedom. However, chi-square is sensitive to sample size and tends to reject reasonable models if the sample is large (Van de Schoot et al., 2012). Therefore, other fit indices, including the comparative fit index (CFI), Tucker-Lewis index (TLI), root mean square error of approximation (RMSEA), and weighted root mean square residual (WRMR) were also taken into account. An adequate fit was considered when the CFI and TLI values were >0.90, while values >0.95 indicated a good fit (Hu and Bentler, 1999). For the RMSEA, values between 0.05 and 0.08 indicate acceptable fit, while values <0.05 indicate good fit (Hu and Bentler, 1999). For the WRMR, values below the cutoff value of 1.0 show good fit (Distefano et al., 2018).

#### Stage 2: Measurement Invariance

Measurement invariance tests were conducted using the strategy described by Meredith and Teresi (2006). The first step was called configural invariance. In this step, the same factor structure had the best fit for each gender group. The second step test for metric invariance, where factor loadings were set item by item to be equal across genders (Van de Schoot et al., 2012) and for the third step – scalar invariance, item intercepts were set invariant across groups. These steps are naturally subsequent, and researchers normally stop testing when any of them produces evidence of non-invariance. Generally speaking, configural, metric, and scalar invariance is enough for implementing MI (Meredith and Teresi, 2006; Rens et al., 2012). For tests of invariance, the changes of CFI (ΔCFI) were used as indices. ΔCFI equal to or <0.01 indicates strong invariance (Cheung and Rensvold, 2002; Sass, 2011).

#### Stage 3: Criterion Validity

To measure the overall psychological condition and whether constructs were related to other criteria, the YPI-CV’s total score and external criteria were correlated. This study used observed variables and examined relations among constructs. All models were performed by Mplus 7.4 (Muthén and Muthén, 1998–2015).

## Results

### Factor Structure

The results of the fit indices are presented in Table 2. The one-factor model and three-correlated-factor model failed to fit the data well (Supplementary Data Sheet S1). At item level (see Table 3), the best-fit model was the bifactor model, with each item loaded on their specific factor as well as the General factor (CFI = 0.964, TLI = 0.953, RMSEA = 0.039, confidence interval [0.028, 0.050], WRMR = 0.815). Six items loaded on the Grandiose-Manipulative factor, with items 1, 4, and 6 having higher loadings (0.405–0.629) on the specific factor than on the General factor (0.292–0.394). Items 2, 3, and 5 loaded only onto the General factor (0.479–0.717). Another six items loaded on the CU factor, with three items (7, 11, and 12) having higher loadings (0.229–0.404) on the specific factor than on the General factor. Although loaded on the specific factor, Item 8 had higher loadings on the General factor. Finally, six items loaded on the Impulsive-Irresponsible factor, with items 13, 14, 16, and 17 have higher loadings on the specific factor than on the General factor. Item 15 only loaded on the specific factor (0.667), while Item 18 only loaded on the General factor (0.612). Details of item loadings are presented in Table 3.

**TABLE 2:** Goodness-of-fit indices for the three tested models of the YPI-CV.

**Model**	**WLSMVχ^2^**	***df***	**CFI**	**TLI**	**RMSEA (90% CI)**	**WRMR**
Single-factor	381.81^∗^	135	0.874	0.844	0.078 [0.069, 0.087]	1.361
Three-factor	374.31^∗^	132	0.860	0.836	0.077 [0.069, 0.086]	1.347
Bifactor	184.03^∗^	117	0.964	0.953	0.039 [0.028, 0.050]	0.815

*WLSMV = weighted least squares with mean and variance adjustment; *df* = degree of freedom; TLI = Tucker-Lewis index; CFI = comparative fit index; RMSEA = root mean square error of approximation; 90% CI = 90% confidence interval; WRMR = weighted root mean square residual. ^∗^*p* < 0.05.*

**TABLE 3:** Factor loading based on bifactor model for the YPI-CV.

**Item**	**Grandiose-manipulative**	**Callous-unemotional**	**Impulsive-irresponsible**	**General psychopathy**
1. Con people than most other people	0.405^∗∗^			0.139
2. Getting people to believe	0.055			0.717^∗∗^
3. Far beyond other’s talent	0.176			0.537^∗∗^
4. Manipulate people	0.432^∗∗^			0.394^∗∗^
5. Use others	0.158			0.479^∗∗^
6. Become a well-known person	0.629^∗∗^			0.292^∗∗^
7. Crying is a sign of weakness		0.281^∗∗^		0.555^∗∗^
8. Should not help people		0.945^∗∗^		0.343^∗∗^
9. Nervous and worried		0.099		0.458^∗∗^
10. Don’t understand touching		0.080		0.719^∗∗^
11. Feel guilty and remorseful		0.404^∗∗^		0.485^∗∗^
12. Don’t let feelings affect		0.229^∗^		0.286^∗∗^
13. Skipped school or work			0.619^∗∗^	0.429^∗∗^
14. Impulsive			0.621^∗∗^	0.517^∗∗^
15. Talk first and think later			0.667^∗∗^	0.135
16. Get bored quickly			0.468^∗∗^	0.226^∗∗^
17. Do things without thinking ahead			0.395^∗∗^	0.522^∗∗^
18. Borrow something and lost it			0.075	0.612^∗∗^

*^∗^*p* < 0.01; ^∗∗^*p* < 0.001.*

### Measurement Invariance

Because the bifactor model was the best fitting model of all the competing models, the MI was examined based on this model. Results showed that the configural invariance model (M0) fit the data very well (RMSEA = 0.045, TLI = 0.934, CFI = 0.942; see Table 4), and most factor loadings were significant (*p_*s*_* < 0.05). The metric invariance model (M1) was tested with factor loading constrained to be equal across groups, and results showed a good model fit (RMSEA = 0.043, TLI = 0.939, CFI = 0.947). M1 was not significantly different from the M0 (ΔCFI = 0.005), indicating that factor loadings were invariant across genders. When thresholds and factor loadings were constrained to be equal across groups (M2), results showed a good model fit (RMSEA = 0.038, TLI = 0.953, CFI = 0.953). Compared to the M1, M2 was not significantly different (ΔCFI = 0.006), indicating that scalar invariance can hold across gender. Overall, MI between male and female children was demonstrated, indicating that gender comparisons can be meaningfully made for the YPI-CV.

**TABLE 4:** Fit indices for measurement invariance.

**Bifactor**	**WLSMVχ^2^**	***df***	***p***	**Diff test**	**CFI**	**TLI**	**RMSEA**	**ΔCFI**
M0 – configural model	370.872^∗^	270	0.000	–	0.942	0.934	0.045	–
M1 – loading	360.336^∗^	268	0.001	–	0.947	0.939	0.043	0.005
M2 – threshold	382.764^∗^	302	0.001	0.0498	0.953	0.953	0.038	0.006

*WLSMV = weighted least squares with mean and variance adjustment; *df* = degree of freedom; Diff test = difference test based on χ^2^; TLI = Tucker-Lewis index; CFI = comparative fit index; RMSEA = root mean square error of approximation; ΔCFI = change in comparative fit index relative to the preceding model; ^∗^*p* < 0.05; chi-square difference test with WLSMV estimation is different from the conventional chi-square difference test.*

### Criterion Validity

Low to moderate correlations were found between the YPI-CV total score and several criteria variables. More specifically, the YPI-CV total score was significantly associated with the SDQ difficulties score (excluding the prosocial subscale; *r* = 0.403, *p* < 0.01), but not the two SCPV subscales (*p* > 0.05). The correlation between the YPI-CV total score and the INS factor of the CPTI was 0.27 (*p* < 0.05). Neither of the other two subscales of the CPTI was correlated to the total score of the YPI-CV (see Table 5).

**TABLE 5:** Zero-order correlations between YPI-CV scores and external variables.

**Name of scales**	**1**	**2**	**3**	**4**	**5**	**6**
1. YPI-CV						
2. Grandiose- deceitful	0.180					
3. Callous-unemotional	0.164	0.773^∗∗^				
4. Impulsive-need for stimulation	0.270^∗^	0.643^∗∗^	0.681^∗∗^			
5. Emotion regulation	–0.060	–0.245^∗∗^	–0.357^∗∗^	–0.211^∗∗^		
6. Prosocial/communication skills	0.051	–0.375^∗∗^	–0.533^∗∗^	–0.271^∗∗^	0.706^∗∗^	
7. SDQ total difficulties score	0.288^∗∗^	0.393^∗∗^	0.465^∗∗^	0.417^∗∗^	–0.328^∗∗^	0.341^∗∗^

**^∗^p* < 0.05; ^∗∗^*p* < 0.01.*

## Discussion

The primary purpose of this study was to examine the psychometric properties and MI of the YPI-CV in a sample of Chinese children. At the item level, the bifactor model of the YPI-CV fits the data best. In addition, the factor structure of the YPI-CV was equivalent across male and female children. Finally, the correlations between the YPI-CV total score and external variables were replicated and extended prior findings to add support for the convergent and discriminate validity of the YPI-CV. Although the reliability of the YPI-CV total score was acceptable, the reliabilities for the three subscales were low.

### Factor Structure

The present study compared a one-factor model, a three-correlated-factor model, and a bifactor model of the YPI-CV (Poythress et al., 2006; Patrick et al., 2007; Pihet et al., 2014; Wang et al., 2017). Findings showed that both the one-factor and three-correlated-factor models failed to fit the data well, although the one-factor model seemed to fit better than the three-correlated-factor model. As expected, the bifactor model fit the data very well, in which all 18 items of the YPI-CV loaded significantly on the general psychopathy factor, along with the three separate uncorrelated dimensions. In line with findings in a Dutch sample (Zwaanswijk et al., 2017), our results provide further support for the latent bifactor structure of the youth psychopathy in Chinese children.

If the one-factor model or three-correlated-factor model fit better than the bifactor model, this could suggested that the dimensions may share a pathway through a common process, and could be explained by genetic factors (Tuvblad et al., 2014). In contrast, we found that the bifactor model fits the data best. The bifactor model is very beneficial when a domain-general factor and similar independent contents coexist (Reise et al., 2007), and change the way about scoring individuals on multiple correlated subscales. Under a bifactor framework, we can be interested in a general construct and several specific domains simultaneously. Other instruments assessing psychopathy also showed that the bifactor model is better than other models, such as PCL-SV (Patrick et al., 2007) and Hare Self-Report Psychopathy Scale (Debowska et al., 2014). These findings may have important implications for research on etiologic mechanisms contributing to the syndrome of psychopathy. Specifically, the bifactor model implies that the general psychopathy factor and three uncorrelated dimensions may affect the etiological process differently, suggesting that psychopathy may be a compound rather than a multifaceted trait (Benning et al., 2003; Pechorro et al., 2015).

### Measurement Invariance

Many studies have compared groups according to their observed scores, but differences may arise from measurement bias between groups. For instance, if the MI does not hold across gender groups, observed different scores between males and females might not be directly comparable (Byrne et al., 1989; Gregorich, 2006). Thus, it is essential to take MI into account when conducting cross-group research. Our findings suggest that the YPI-CV demonstrates metric MI across gender. Metric invariance entails equal factor loadings for items across groups and suggests that items share equivalent meaning in terms of their relationship to the factor across groups.

### Reliability and Criterion Validity

The internal consistency for the YPI-CV total score is acceptable, while the results of factor scores are unacceptable. This finding suggests that when we use the YPI-CV, the total score is more reliable than the factor scores, at least for 9- to 12-year-old Chinese children. Similarly, in terms of α, the internal consistency for the YPI-SV scores was lower than that for the YPI in Chinese adolescents (Wang et al., 2017). Lower reliability may be partly due to its short length (Cureton, 1958) as well as the sample size (Fitzpatrick and Yen, 2001). The alpha values are lower for the child self-report version than for the adolescent self-report versions. These might be due to the fact that children younger than 12 years have limited reading comprehension (e.g., Soto et al., 2008) which could further influence consistent responses on items of the YPI-CV.

A moderate correlation between the YPI-CV total score and SDQ total difficulties score was found, consistent with findings by Baardewijk et al. (2011). In addition, we found significant correlations between parent-reported problem behavior (SDQ; Goodman, 1997) and the YPI-CV, with *r* = 0.45 (*p* < 0.01). For the SCPV scale, neither the total score nor the two subscale scores correlated with the YPI-CV total score at 0.05 level. Lower correlation between criteria variables and the YPI-CV total scores may be caused by different raters, as the YPI-CV was self-reported and parent evaluated criteria variables (such as the CPTI and SCPV). Finally, the CPTI and YPI-CV are two similar but different instruments, which were positively correlated, overlapping to some extent, providing information from different aspects.

### Limitations

The present study has several limitations. First, according to Blair et al. (2005), in the general population, a clinical diagnosis of psychopathy is quite rare (<1%). It restricts the range in psychopathy scores in our non-clinical sample, thereby possibly underestimating the reliability of YPI-CV, also the relations between psychopathy and other variables. Future studies with a clinical sample may help us. Second, the external criteria of the YPI-CV were found to be limited in the present study. Other criteria relevant to the psychopathy concept should be considered in future studies, such as aggression (Forth and Book, 2010) and emotional intelligence (Megías et al., 2018). Third, the lower reliability of some criteria variables, such as SDQ (Janni et al., 2012), was not high. Lower reliability may attenuate correlations between criteria measures and variables of interest (Lachin, 2004). Finally, the present study was conducted in a sample of school children; future research should include clinical samples with a higher prevalence of psychopathic traits and conduct problems.

The results of the present investigation indicate that the bifactor model better represented the underlying structure of the YPI-CV in Chinese children sample. Furthermore, the bifactor structure was shown to be invariant across genders. The total score of the YPI-CV has acceptable reliability; however, the reliabilities of the three subscales were unacceptable. In conclusion, the current study suggests that the bifactor model of the YPI-CV should be used when examining relationships with outcome variables, but with a focus on the total score of the YPI-CV. However, in these situations, the factor scores of the YPI-CV should be reported with caution, especially with a non-clinical sample.

## Data Availability Statement

The supplementary material datasheet of this article can be downloaded via doi: 10.6084/m9.figshare.10431365.

## Ethics Statement

All the research procedures met the ethical guidelines of the American Psychological Association and were approved by the Institutional Review Board (IRB) at Guangzhou University. Written informed consent to participate in this study was provided by the participants’ legal guardian/next of kin.

## Author Contributions

FR, Q-PZ, and JL made substantial contributions to the analysis and interpretation of the data, drafted the manuscript, provided final approval of the version to be published, and agreed to be accountable for all aspects of the work in ensuring that questions related to the accuracy or integrity of any part of the work were appropriately investigated and resolved. ML, JD, XZ, and M-CW helped out in the interpretation of the data for the work, revised the manuscript critically for important intellectual content, provided final approval of the version to be published, and agreed to be accountable for all aspects of the work in ensuring that questions related to the accuracy or integrity of any part of the work are appropriately investigated and resolved.

## Conflict of Interest

The authors declare that the research was conducted in the absence of any commercial or financial relationships that could be construed as a potential conflict of interest.

## Footnotes

1 http://www.sdqinfo.com

## Supplementary Material

DATA SHEET S1 Raw data for the study.
